# Primary Lamellar Macular Holes: To Vit or Not to Vit

**DOI:** 10.3390/jcm11175046

**Published:** 2022-08-28

**Authors:** Lihteh Wu, Ryan Bradshaw

**Affiliations:** 1 Asociados de Macula, Vitreo y Retina de Costa Rica, Primer Piso Torre Mercedes, Paseo Colón, San José 10102, Costa Rica; 2 Illinois Eye and Ear Infirmary, Department of Ophthalmology, School of Medicine, University of Illinois Chicago, Chicago, IL 60607, USA; 3 Centro de Oftalmologia y Microcirugia Boyd, Departamento de Retina, Panama City 0816-02593, Panama

**Keywords:** lamellar macular hole, lamellar hole epiretinal proliferation, epiretinal proliferation, macular pseudohole, vitrectomy, ERM foveoschisis, partial thickness macular holes, macular holes

## Abstract

There is a wide spectrum of macular conditions that are characterized by an irregular foveal contour caused by a break in the inner fovea. These include full-thickness macular hole (FTMH), foveal pseudocyst, lamellar macular hole (LMH) and macular pseudohole (MPH). Clinical examination of vitreomacular interface disorders is notoriously poor in differentiating these conditions. These conditions were initially described with slit-lamp biomicroscopy, and the main goal was to distinguish an FTMH from the others. The introduction of optical coherence tomography (OCT) has revolutionized our understanding of the foveal microstructural anatomy and has facilitated differentiating these conditions from an FTMH. However, the definitions of the other conditions, particularly LMH, has evolved over the past two decades. Initially the term LMH encompassed a wide spectrum of clinical conditions. As OCT became more widely used and observations became more refined, two different phenotypes of LMH became apparent, raising the question of different pathogenic mechanisms for each phenotype. Tractional and degenerative pathological mechanisms were proposed. Epiretinal membranes (ERMs) associated with each phenotype were identified. Typical ERMs were associated with a tractional mechanism, whereas an epiretinal proliferation was associated with a degenerative mechanism. Epiretinal proliferation represents Müller cell proliferation as a reactive process to retinal injury. These two types of ERM were differentiated by their characteristics on SD-OCT. The latest consensus definitions take into account this phenotypic differentiation and classifies these entities into LMH, MPH and ERM foveoschisis. The initial event in both ERM foveoschisis and LMH is a tractional event that disrupts the Müller cell cone in the foveola or the foveal walls. Depending on the extent of Müller cell disruption, either a LMH or an ERM foveoschisis may develop. Although surgical intervention for LMH remains controversial and no clear guidelines exist for pars plana vitrectomy (PPV), eyes with symptomatic, progressive ERM foveoschisis and LMH may benefit from surgical intervention.

## 1. Introduction

There is a wide spectrum of macular conditions that are characterized by abnormalities of the foveal architecture caused by a break in the inner fovea. These conditions include full thickness macular hole (FTMH), foveal pseudocyst, lamellar macular hole (LMH) and macular pseudohole (MPH). Prior to 1991, when Kelly and Wendel [1] pioneered vitrectomy surgery for FTMH, a previously untreatable condition, differentiation between these conditions was mostly of an academic interest. Currently modern FTMH repair with PPV, posterior hyaloid detachment and ILM peeling with gas tamponade and post-operative face-down positioning results in closure of most of the FTMH ≤ 400 um in size [2]. MPH and LMH were initially defined biomicroscopically, and the purpose was to distinguish them from a FTMH [3,4,5]. Gass [3] described the biomicroscopic findings of an eye that developed an inner LMH hole as a result of chronic aphakic cystoid macular edema. Following the patient’s death, the eye was examined histopathologically which confirmed the biomicroscopic findings of an inner LMH. The introduction of optical coherence tomography (OCT), particularly spectral domain (SD) OCT, into routine clinical practice revolutionized our understanding of diseases of the vitreomacular interface and facilitated differentiating these conditions from a FTMH [6,7]. All of these conditions had to be reinterpreted on the basis of OCT findings. It must be borne in mind that the definition of LMH has evolved over the past two decades. Depending on when a particular manuscript was published, different and conflicting definitions, criteria and terminology were used to describe different conditions associated with an abnormal foveal contour. It is not surprising then, that natural history and treatment outcomes vary from study to study [8,9,10,11,12,13]. The purpose of the current review is to update the reader with the current understanding of LMH.

## 2. Historical Background

In 1975 Gass [3] reported the clinical histopathologic correlation of an eye with a well-defined macular reddish oval lesion with a preserved foveal reflex that developed a partial loss of foveal tissue secondary to long standing pseudophakic cystoid macular edema (CME). He chose to use the term LMH to describe these findings. MPH was first described by Allen and Gass [5] in 1976 as a peculiar macular lesion that simulated a macular hole. Its pathogenesis was ascribed to the spontaneous contraction of an epiretinal membrane that surrounded the foveal area. Fluorescein angiography (FA) was not a useful imaging modality to differentiate among these conditions. MPH commonly exhibit early hyperfluorescence within the area of the MPH which may be confused with a FTMH [14]. Time domain OCT is able to differentiate between a MPH and a LMH [8]. In eyes with a LMH, the OCT profile is irregular; the foveal edges are split; and the foveal center is thinner than normal. In contrast, eyes with a MPH are characterized by the presence of a deep foveal pit, verticalized edges and a thickened macula caused by the contraction of the ERM [8]. In 2013 the International Vitreoretinal Traction Study Group defined LMH and MPH based on SD-OCT B scan images [2]. However, it was soon realized that in some instances the differentiation between these conditions was not clear cut [15]. As OCT became more widely used and observations became more refined, two different phenotypes of LMH became apparent: raising the question of different pathogenic mechanisms for each phenotype. In 2016, Govetto et al. [16] proposed sub-dividing LMH into two distinct categories, namely tractional and degenerative LMH. Recently an international panel of vitreoretinal experts recognized that the prevailing definition of LMH encompassed a wide spectrum of conditions characterized by a break in the inner fovea and an irregular contour [17]. They recognized that many different clinical conditions with different pathophysiological were included together. In the hopes of facilitating future research they published an SD-OCT based consensus definition for LMH, ERM foveoschisis and MPH. Govetto et al. [16] tractional LMH became ERM foveoschisis whereas degenerative LMH became LMH [17].

## 3. Epidemiology

The reported prevalence of LMH in different populations has ranged anywhere from 0.1% to 3.6% [18,19,20,21,22]. Most people who develop a LMH are older than 50 years of age [18,19,20]. In the Beaver Dam Eye Study, a population-based study of people aged 63 to 102 years old, participants were assessed with SD-OCT. The prevalence of LMH was estimated to be 3.6%. It was higher in eyes with a history of prior cataract surgery. Age did not influence the prevalence of LMH. After adjusting for age and gender, ERM were associated with the presence of LMH, macular cysts and FTMH [18]. The Maastricht Study was an observational prospective population-based study of individuals aged between 40 and 75 years from the Netherlands. The prevalence of LMH was estimated to be 0.9%. Women were more prone to develop LMH [19]. The Montrachet Study, a French population-based study, reported a prevalence of 1% and 0.4% for LMH and MPH, respectively [20]. A cross sectional study of 2257 healthy Spanish individuals older than 45 years of age that underwent SD-OCT imaging revealed a LMH prevalence of 0.1% [21]. A South Korean study of 698 patients scheduled for cataract surgery with a normal biomicroscopic examination of the macula, underwent pre-operative SD-OCT or swept source OCT. They reported a 0.3% prevalence for LMH [22].

Bilateral LMH appears to be a relatively uncommon occurrence [23]. A retrospective study of 35 individuals with a LMH revealed that in the fellow eye, 83% had a vitreomacular abnormality. However, only 9% of patients had an LMH. The most frequent finding was a tractional ERM seen in 74% of fellow eyes [23].

## 4. Multimodal Imaging

Symptoms of LMH are similar to those found in other vitreoretinal interface syndromes such as ERM, MPH and early FTMH. These conditions were all defined biomicroscopically, and the main goal was to distinguish a FTMH from the other conditions. Typically, patients have a BCVA of ≥ 20/40 [24,25]. Many patients are asymptomatic. Others complain of decreased visual acuity, metamorphopsia and a central scotoma [25]. Clinical examination of vitreomacular interface disorders is notoriously poor in differentiating these conditions. Functional tests such as visual acuity, microperimetry and the Watzke-Allen test are of little help as well [26]. MPH have the clinical appearance of a macular hole but with no loss of foveal tissue. In contrast LMH were defined by a partial tissue loss [2]. Distinguishing among these conditions proved clinically challenging. The introduction of optical coherence tomography (OCT), particularly spectral domain (SD) OCT, has been instrumental in expanding our understanding and differentiation of these conditions from a FTMH. Since prognosis and management differ among these conditions, reliable diagnostic criteria are needed. Biomicroscopic fundus examination was able to diagnose only 28% of LMH diagnosed with time domain OCT [8]. In the Beaver Dam Eye study, only 1.6% of eyes with a SD-OCT diagnosis of a LMH were detected with fundus photographs [18].

However, once OCT, particularly SD-OCT was introduced into routine clinical practice, all of these conditions had to re-interpreted on the basis of OCT findings. Since then, differing definitions and criteria for diagnosing LMH and MPH have evolved over the years. Authors have used different definitions for what they considered to be LMH at the time. In 2004 time domain OCT criteria were defined for MPH and LMH [8]. Eyes with an MPH had a steepened foveal pit whose diameter was small and the foveal edges were thickened. The central foveal thickness was within normal limits or had a slight increase. The perifoveal thickness was increased. In contrast LMH were defined by a thin irregular foveal floor, a thinner central foveal thickness, split foveal edges and near normal perifoveal thickness [8]. In 2006 findings in ultrahigh definition OCT, that included an irregular foveal contour, a break in the inner fovea, intraretinal split and an absence of a full foveal defect with intact foveal photoreceptors, were used to define a LMH [9,27]. In 2013 the International Vitreomacular Traction Study Group defined LMH and MPH according to SD-OCT B scan images. They defined a LMH as an eye with an irregular contour, a defect in the inner fovea, intraretinal splitting and maintenance of an intact photoreceptor layer. Similarly, an eye with a MPH was characterized by an invaginated or heaped foveal edges, concomitant ERM with a central opening, steep macular contour to the central fovea with near normal central foveal thickness and no loss of retinal tissue [2].

OCT demonstrated that ERM are commonly associated with LMH ranging anywhere from 62% to 100% of eyes [8,11,15,28,29]. These are characterized by a highly reflective line immediately anterior to and separate from the retinal nerve fiber layer (RNFL) on SD-OCT. Ref. [11] ERM can exert unidirectional, pluridirectional and concentric tangential lines of traction on the macular surface [30,31]. Asymmetric tangential traction along different directions results in a cleavage of the foveal pit edge whereas symmetric centripetal contraction leads to straight smooth edges on the SD-OCT [31]. It was noted that in eyes with LMH the position of the foveal contour was below the outer plexiform layer whereas in normal eyes it is located at the level of the outer plexiform layer [28]. Since then, other OCT findings like epiretinal proliferation (ERP), ellipsoid zone disruption and intraretinal splitting have been described in these eyes [9,27,28,31].

Despite these OCT based definitions, the differentiation of LMH and MPH is not always straightforward. Several investigators believe that intraretinal splitting is the key differentiator between LMH and MPH [8,11,32]. In contrast others do not consider the presence of intraretinal splitting as part of the diagnostic criteria of LMH [15,31]. In 2012, Michalewska and co-workers [28] reviewed their SD-OCT database of over 10,000 patients and identified 125 eyes from 116 patients with a non-full thickness macular hole. All the eyes had a co-existing ERM. Based on morphological findings on the SD-OCT, they subdivided the eyes into 4 subgroups: MPH, Para-LMH, MPH with lamellar defects and LMH. Based on the observation that 40% of eyes had different subtypes present in the same eye, they concluded that all these subtypes were different phenotypes of the same progressive condition. In addition eyes with LMH were associated with outer retinal disruptions calling into question the assumption that the photoreceptors are intact in LMH [28]. Furthermore, in several cases of MPH, progressive ERM contraction led to a LMH [28,33].

In Michalewska’s series, 60% of eyes had a declining visual acuity with a mean loss of 2.4 lines of Snellen after a mean follow-up of 14 months. Photoreceptor layer defects either appeared or enlarged in 36% of eyes. The outer diameter of the foveal defect increased in 33% of eyes [28]. These cases lend support to Gaudric et al.’s [31] observation that eyes with an intraretinal split along a foveal edge should not be classified as LMH but as a variant of MPH. In these eyes SD-OCT revealed that some eyes exhibit an incomplete lamellar cleavage between the inner and outer retina of their edges. Stretched Henle fibers still connected the inner and outer retina [31]. In contrast other authors classified these eyes as LMH [11,32].

In 2016 Govetto and collaborators [16] upon review of the SD-OCT images of their 102 consecutive eyes diagnosed with LMH, concluded that two distinct clinical entities, degenerative and tractional LMH, formed part of the spectrum of what was then defined as an LMH. According to them, tractional LMH is characterized by a schitic separation of the neurosensory retina between the outer nuclear layer and the outer plexiform layer. In contrast, degenerative LMH is characterized by intraretinal cavitations that can affect all retinal layers, non-tractional epiretinal proliferation (ERP) and a retinal bump. Underscoring the difficulty in classifying these eyes into specific categories, 11 eyes had mixed characteristics [16].

**Figure 1:**
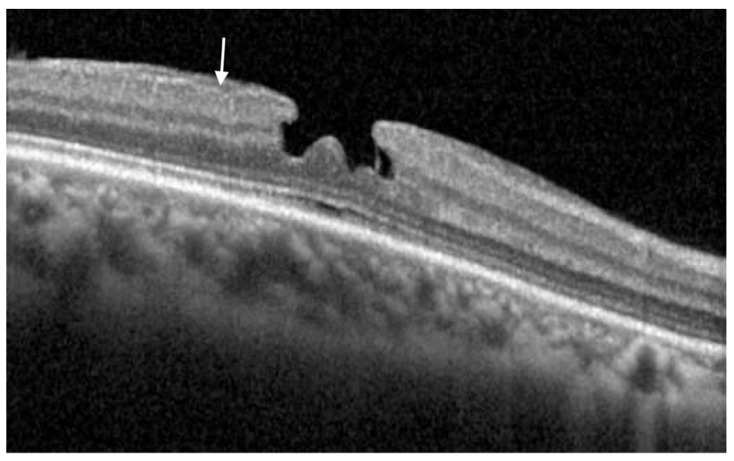
B scan SD-OCT of an eye with an LMH. Notice the epiretinal proliferation (arrow) that is isoreflective and conforms to the retinal surface without altering the retina shape. There is no evidence of traction. The foveal contour is irregular. SD-OCT: Spectral Domain optical coherence tomography; LMH: Lamellar Macular Hole.

**Figure 2:**
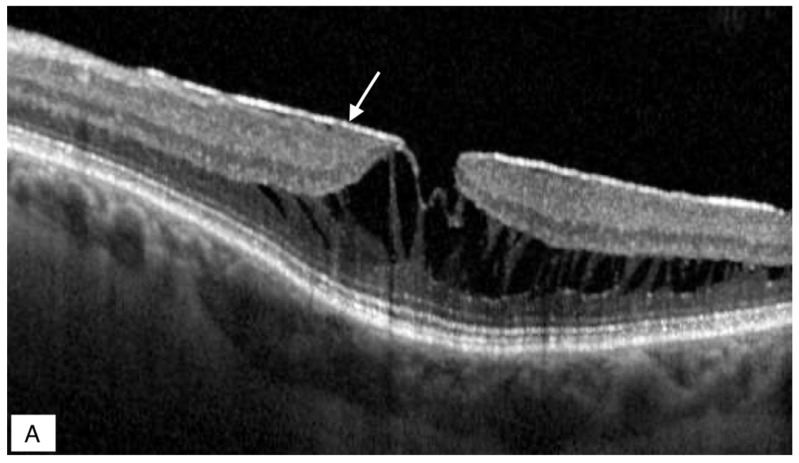

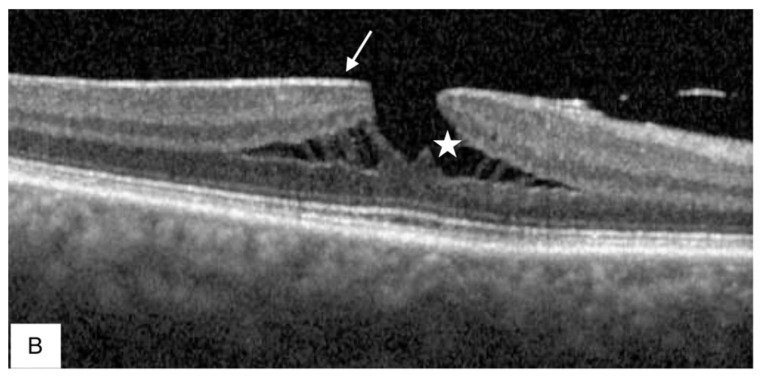
(**A**) scan SD-OCT of an eye with ERM foveoschisis. The arrow points to a hyperreflective line at the vitreo-macular interface. It represents an ERM. Notice the intraretinal hyporeflective spaces representing the foveoschisis at the level of Henle’s layer. (**B**) B scan SD-OCT of an eye with ERM foveoschisis. The arrow points to a hyperreflective line that is an ERM. Notice the foveoschisis (star). SD-OCT: Spectral Domain optical coherence tomography; ERM: epiretinal membrane.

In 2020 an international panel of vitreoretinal experts recognized that the prevailing definition of LMH encompassed a wide spectrum of conditions characterized by a break in the inner fovea and an irregular contour. They felt that many different clinical conditions with different pathophysiological mechanisms were lumped together. In the hopes of facilitating future research, they published an SD-OCT based consensus definition for LMH, ERM foveoschisis and MPH [17]. Mandatory criteria for a LMH included an irregular foveal contour; a foveal cavity with undermined edges and the presence of at least one other sign of foveal tissue loss. In order for an edge to be considered undermined, the angle between the retinal surface and the edge of the hole on the B scan OCT has to be <90°. Optional criteria included the presence of epiretinal proliferation, the presence of a central foveal bump and disruption of the ellipsoid zone. A foveal bump was defined as *“a bulge of retinal tissue in the center of the fovea, usually surrounded by foveal cavities with undermined edges.”* [17]. (Figure 1) This definition of LMH is reminiscent of Govetto et al.’s [16] definition for degenerative LMH. Mandatory criteria for an ERM foveoschisis included the presence of an ERM and the presence of foveoschisis at the level of Henle fiber layer. An ERM was defined as an irregular and hyperreflective layer over the ILM. The underlying retina may express signs of wrinkling such as the presence of hyporeflective spaces between the ILM and the ERM. Optional criteria for ERM foveoschisis included the presence of microcystoid spaces in the inner nuclear layer, an increase in retinal thickness and the presence of retinal wrinkling [17]. The description of ERM foveoschisis resembled the tractional LMH definition of Govetto et al. [16] (Figure 2) Mandatory criteria for an MPH included the presence of a foveal sparing ERM; presence of a steepened foveal profile and an increased central retinal thickness. Optional criteria included microcystoid spaces in the inner nuclear layer and a normal retinal thickness [17].

Despite the capability of SD-OCT to visualize the macula in detail, it may fail to detect very small losses of foveal tissue [34]. Blue fundus autofluorescence (FAF) is more sensitive than SD-OCT in detecting these small changes. The main FAF signal is derived from the lipofuscin in the RPE. In the fovea this signal is attenuated by the presence of the luteal pigment [35]. LMH exhibit an increased signal of blue FAF [34]. Unfortunately, blue FAF cannot discriminate among LMH, ERM foveoschisis and MPH since all of these conditions exhibit an increased blue FAF signal at the fovea [36]. This hyper blue FAF is caused by either an actual loss of foveal tissue or a centrifugal displacement of foveal tissue containing macular pigment [37,38]. Italian investigators compared the blue FAF and the SD-OCT findings in eyes with LMH. They reported a strong correlation between the LMH diameter measured by blue FAF and the SD-OCT measured at the level of the outer plexiform layer [39].

En face SD-OCT imaging and multicolor imaging, which uses a confocal scanning laser ophthalmoscope to obtain near infrared reflectance (NIR), green reflectance and blue reflectance en face images, allows visualization of the entire extent and the vectorial directions of the tractional elements acting on the macula. Contraction epicenters are easily observed under en face imaging [30,31,40]. Epimacular traction can be characterized as unidirectional, pluridirectional or concentric. Unidirectional traction is characterized by folds pulling towards a non-foveal center of contraction whereas pluridirectional traction consists of multiple contraction centers with multiple directions of traction. In contrast, concentric traction consists of folds pulling towards the center of the fovea [30]. ERM contraction is responsible for these morphological changes [40]. En face OCT revealed that the intraretinal splitting occurs within the outer plexiform layer [29]. NIR imaging revealed retinal folds in tractional LMH. No folds were seen in degenerative LMH in NIR imaging [30].

Several SD-OCT biomarkers have been explored as visual acuity predictors. The status of the foveal microstructure, namely the external limiting membrane (ELM) and the ellipsoid zone (EZ), correlates with the central retinal sensitivity and the BCVA [15,41]. In a prospective observational study of 54 patients with LMH, 26% of eyes had the ELM and the ellipsoid zone disrupted. In these eyes the BCVA and the central retinal sensitivity were significantly poorer [41]. They noted that 29% of the eyes with a LMH had photoreceptor layer defects. Photoreceptor layer defects, maximum retinal thickness and the outer diameter of the foveal defect correlate with visual acuity [28]. En face SD-OCT imaging allows quantification of intraretinal splitting within the outer plexiform layer. In this retrospective study of 42 eyes, the area of intraretinal splitting did not correlate with visual acuity. However, disruption of the EZ was correlated with visual loss. EZ disruption correlated with the area of splitting [29].

Tractional and degenerative LMH appear to have different macular microvascular parameters as studied by OCTA [42]. Eyes with tractional LMH exhibit a smaller foveal avascular zone (FAZ) area, a higher foveal vascular density (VD) and a lower parafoveal VD in both the superficial capillary plexus (SCP) and the deep capillary plexus (DCP) than control eyes and eyes with degenerative LMH. Eyes with a degenerative LMH had lower parafoveal VDs in both the SCP and the DCP. Furthermore, the size of the VD was correlated with the BCVA in these eyes [42]. Catania and colleagues [43] compared OCTA parameters in eyes with LMH that progressively lost tissue to those which remained stable. Eyes with progressive tissue loss manifested decreased foveal VD in the SCP, and decreased perfusion density in both the SCP, DCP and parafoveal areas.

One of the major limitations of our current imaging techniques is that none can reliably determine the presence of actual retinal tissue loss.

## 5. Epiretinal Proliferation (ERP)

**Figure 3:**
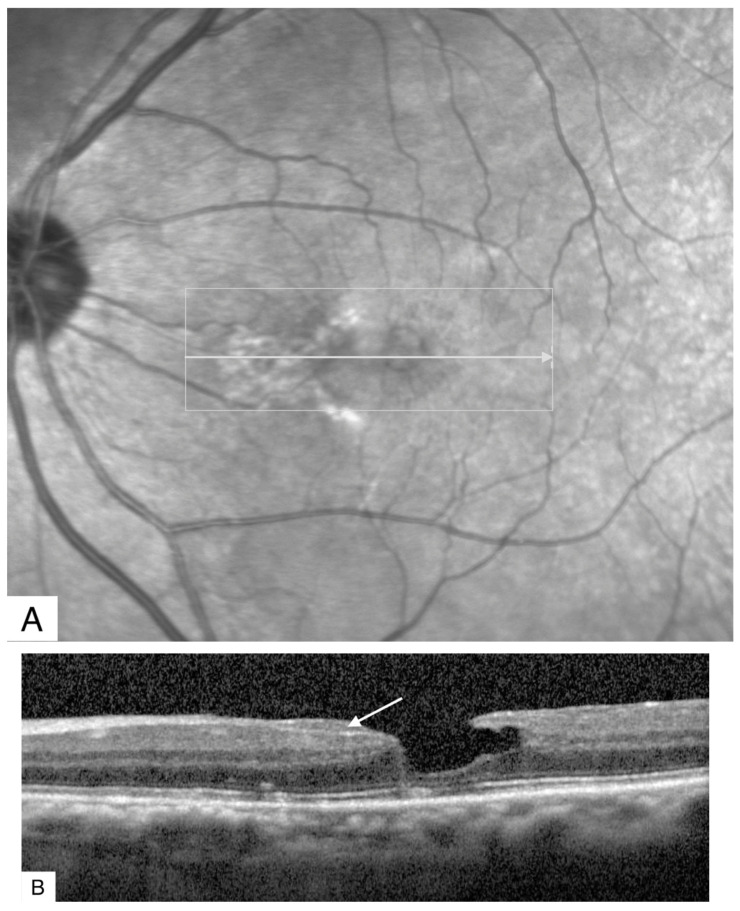
(**A**) Infrared reflectance imaging of an eye demonstrating alterations in the fovea. The horizontal arrow shows the direction of the SD-OCT scan. (**B**) SD-OCT scan showing epiretinal proliferation (arrow) that is isoreflective and conforms to the retinal surface without altering the retina shape. There is no evidence of traction. The foveal contour is irregular. SD-OCT: Spectral domain optical coherence tomography.

In 2006 Witkin et al. [9] described ERM with an unusual thick appearance in eyes with LMH. These were described as moderately reflective material that filled the space between the RNFL and the inner border of the ERM. The authors of this observation speculated that trapped vitreous or posterior hyaloid were responsible for this ERM appearance and their presence helped stabilize the macula [11]. In 2011 Parolini et al. [27] further described these “unusual dense non-tractional ERM” and made a point of distinguishing them from the typical tractional ERM. These atypical dense non-tractional ERM followed and conformed to the retinal surface without altering the retinal shape. Furthermore, there were no signs of traction. (Figure 3) It remained unclear whether these atypical ERM represented different disease entities or different stages of the same disease entity [27]. In 2013, Bottoni and associates [44] reported on his cohort of 34 patients that were followed prospectively with serial SD-OCT imaging. Eyes with a “thicker” ERM had a reduced BCVA and a thinner fovea at baseline when compared to eyes with a typical ERM. Based on these observations they suggested that different pathophysiological mechanisms were likely [44]. Additionally, in 2013, Shiraga and associates [45] observed that the ERM in LMH often contained macular pigment and originated from within the LMH. In 2014 Pang and co-workers [46] named this tissue, lamellar hole-associated epiretinal proliferation (LHEP) and defined it as a homogeneous epiretinal material of medium reflectivity on SD-OCT imaging. They noted that this ERP did not distort the ILM and was contiguous with the middle retinal layers and seemed to originate from within the associated retinal defect. In addition LHEP did not manifest contractile properties [46]. Some investigators proposed that the presence of LHEP was a distinct clinical entity and identified a particular subtype of LMH [44,46,47,48]. In 2016 Govetto and co-workers [16] introduced the concept of tractional vs. degenerative LMH. Although LHEP was associated with the degenerative subtype of LMH, the mere presence of LHEP was not sufficient to determine a subtype of LMH. In 2017 Dell’Omo and colleagues [49] suggested that LHEP exerted tangential traction via the ILM.

Although initially associated with LMH, this epiretinal proliferation has also been described in other conditions like FTMH, ERM and other inflammatory, degenerative and vascular diseases [48,50]. Up to 25% of eyes with a FTMH demonstrate the presence of LHEP [46,51]. Since conditions other than LMH have been associated with LHEP, we and others favor the use of the term of epiretinal proliferation (ERP) rather than LHEP [17,52].

Depending on the case series, anywhere from 20% to 53% of eyes with a LMH have ERP associated with it [15,46,49,53,54,55]. In one retrospective case series of 84 eyes, most eyes that exhibited ERP had an associated ERM to it [49].ERP is typically not visible by biomicroscopic examination or color fundus photography. Hyporeflectance on blue reflectance imaging delineates the macular surface that is covered by the ERP [56]. Intraoperative observations by several surgeons describe a yellowish dense tissue with fluffy consistency in the surgically removed ERP [27,45,46,57]. This yellowish pigment was identified as carotenoids that typically form part of the macular xanthophyll pigments [58].

Some studies report that the presence of this ERP correlates with greater photoreceptor layer defects, greater LMH external diameters, a thinner floor and worse baseline visual acuity as compared to eyes without ERP [9,27,44,49,55,59,60,61,62]. Despite these different morphological characteristics, it appears that once the LMH forms with or without ERP, the LMH remains relatively stable. ERP does not accelerate LMH progression [44,47].

The origin of the ERP has been debated extensively. However, due to the limited number of histopathological studies and their small sample sizes, the origin of ERP remains unclear. An immunocytochemical study compared the ultrastructural characteristics of the surgically removed tissue in LMH to MPH. The specimens from LMH contained glial fibrillary acidic protein (GFAP) and hyalocyte markers (collagen type I and III). In contrast the specimens from MPH contained α−smooth muscle actin and GFAP. At the cellular level, LMH specimens consisted of fibroblasts and hyalocytes whereas myofibroblasts predominated in MPH. Based on these observations, the authors concluded that this peculiar ERP is a premacular vitreous remodeling that originates from the vitreal hyalocytes. The cellular origin of ERP was attributed to vitreal hyalocytes [27]. However, Son et al. [57] have criticized the above study stating that the tissue sent for histopathological study did not differentiate between ERP, ILM or ERM. In their own study they carefully surgically removed ERP following ILM removal to avoid contamination of the sample with ILM or ERM. Their surgical specimen reacted strongly with a pankeratin (AE1/AE3) antibody suggesting the involvement of the RPE. Furthermore, they noted that their specimen did not react with antibodies to S-100, a marker of Müller cells. They suggested that ERP was due to RPE proliferation that migrated through ellipsoid zone defects [57]. In a more recent study these same authors stated that the pankeratin AE1/AE3 are not specific for the RPE and could also represent activated Müller cells. In a clinicopathological correlation, Pang et al. [63] reported that the surgical specimen stained with anti-GFAP and anti-glutamine synthetase, a Müller cell specific marker. They attributed the cellular origin of LHEP to activated Müller cells [46,47]. ERP may represent a reparative attempt by Müller cells to deal with retinal insults [17,50,63,64].

## 6. Pathogenesis

To fully understand the morphological changes observed in the SD-OCT, it is necessary to understand the anatomy of the fovea. Müller cells extend for almost the entire retinal thickness and provide the main structural support for the retina. The external limiting membrane (ELM) is formed by the junctional complexes between the photoreceptors and the Müller cells. With the exception of the fovea, the Müller cells course perpendicularly through the retinal thickness [65].

Up until fifty years ago, the human central fovea (central foveal bouquet) was initially thought to only contain cone photoreceptors [66,67]. In 1969 an electron microscopy study of human autopsy eyes revealed that specialized Müller cells were also present in the central fovea and extended from the external limiting membrane to the ILM. Thirty years later Gass [68] expanded upon this hypothesis and proposed the presence of a central plug of specialized inverted cone shaped Müller cells that he named the Müller cell cone. In the center of the fovea, the ELM is discontinuous due to the absence of junctional complexes. Other investigators noted that the floor of the foveola was formed by the Müller cell cone [65,69]. Müller cell cones stabilize the central fovea by binding the cones together in the foveola lending structural support to them [67,68,70]. Disruption of the Müller cell cone transmits stress to the central fovea and may lead to FTMH and LMH formation.

Parafoveal Müller cells are characterized by a z shaped anatomical configuration. The horizontal portion of the z shaped Müller cell forms part of Henle’s fiber layer and the cell processes extend eccentrically towards the center of the fovea [71]. Henle’s fiber layer (HFL) is a structural weak point of the retina. The retina typically and preferentially splits along HFL in tractional conditions such as myopic traction maculopathy, ERM foveoschisis and LMH [29,72].

Recently a central, vertical, hyperreflective line extending from the ILM to the ellipsoid zone was observed on SD-OCT in 50% of eyes prior to the development of a FTMH. In addition, this same finding was seen in 50% of eyes after macular hole closure following PPV and concurrently in 25% of eyes with a LMH. This vertical hyperreflective line may represent vitreomacular traction and serve as a very early marker of FTMH and LMH development [73].

Most cases of ERM foveoschisis arise in eyes without prior macular disease from an abortive process in the formation of a FTMH or as a sequelae of VMTS [11,24,25,28,74]. Initially a posterior vitreous detachment starts to evolve and leads to vitreomacular separation and persistent vitreofoveal adherence leads to a foveal pseudocyst in the inner retina. A foveal pseudocyst has been identified by several researchers as the first step in macular hole formation [24,75,76,77]. Persistent antero-posterior traction may lead to a foveal detachment and a FTMH. If the antero-posterior traction is released prematurely and avulses the inner wall of the foveal cyst a LMH may result instead [24,25,71,77]. The presence of a pseudo-operculum in eyes with LMH serves as further evidence of anteroposterior traction [8]. In these cases, antero-posterior traction leads to foveolar Müller cell cone disruption with elevation of the inner layers of the foveal walls which leads to a schitic splitting between the OPL and HFL [64].

**Figure 4:**
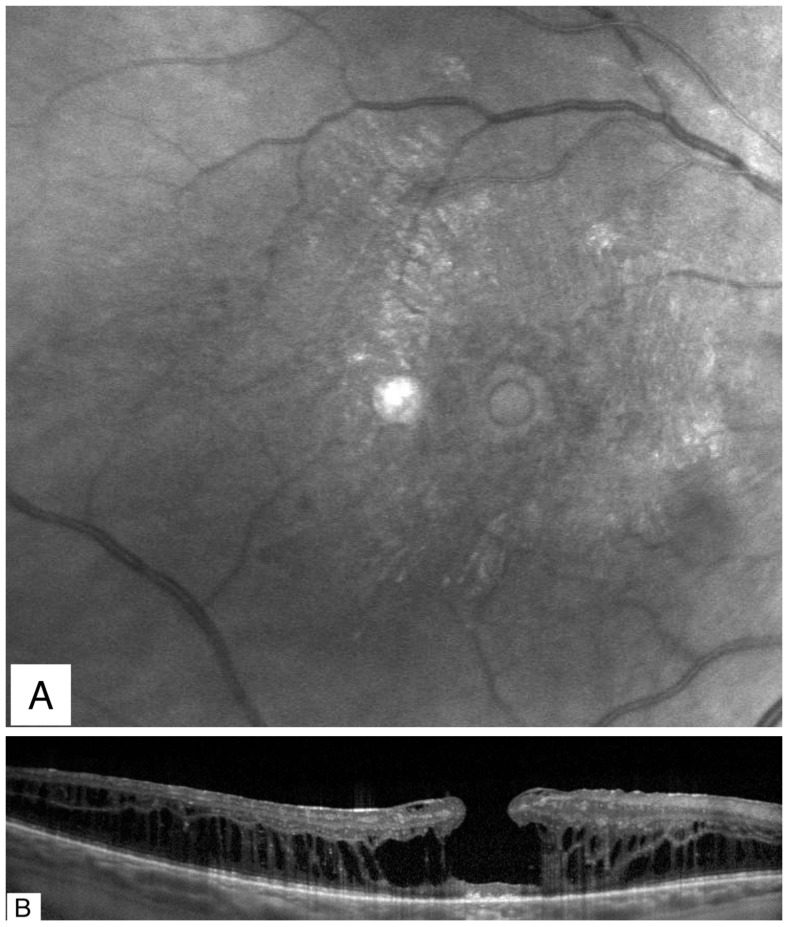
A 65-year-old woman with long-standing diabetic macular edema which led to a secondary LMH. The visual acuity was counting fingers. (**A**) Infrared reflectance imaging showing a round punched-out lesion in the center of the macula simulating a full thickness macular hole. (**B**) Spectral domain optical coherence tomography demonstrating residual tissue at the foveal floor confirming the presence of a secondary LMH rather than a FTMH. LMH: lamellar macular hole; FTMH: full thickness macular hole.

**Figure 5:**
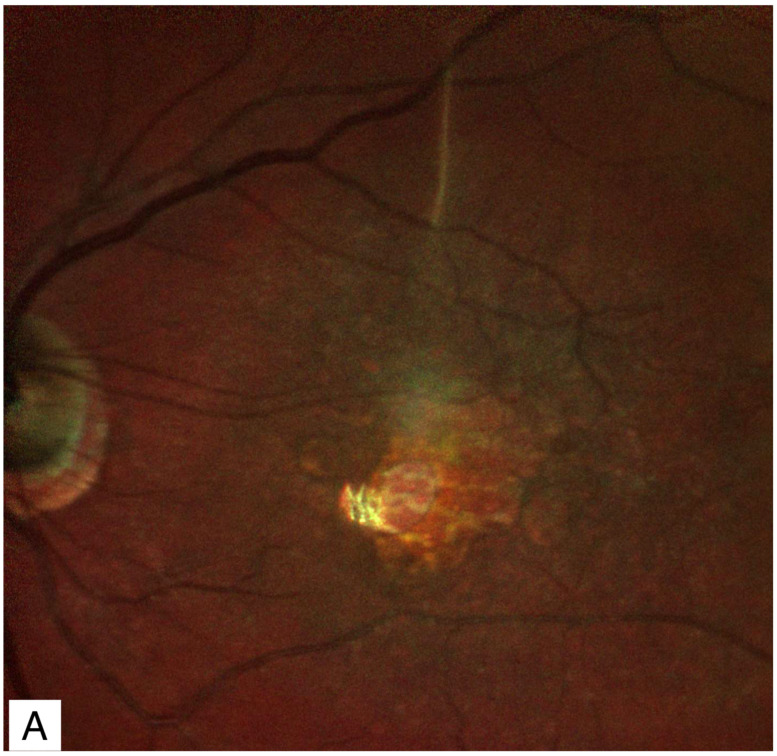

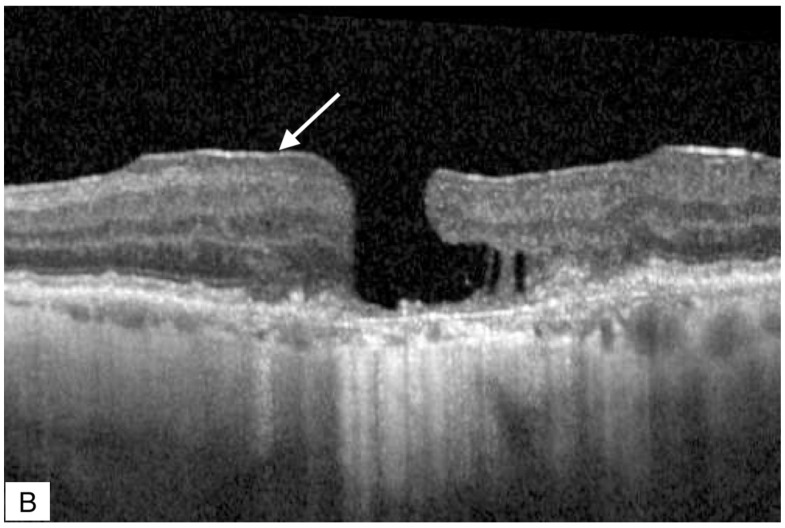
A 96-year-old man with long standing macular degeneration. The visual acuity was counting fingers. (**A**) Multicolor reflectance image showing some drusen and an irregular patch of central atrophy. (**B**) Spectral domain optical coherence tomography demonstrating residual tissue at the foveal floor confirming the presence of a secondary LMH. The arrow points to an ERM. LMH: lamellar macular hole; ERM: epiretinal membrane.

Eyes with conditions complicated by chronic CME such as the Irvine-Gass syndrome [3,4], diabetic macular edema [78], (Figure 4) retinal vein occlusions [78,79,80,81,82], retinochoroiditis [83], oculocutaneous albinism [84], age-related macular degeneration [85,86], (Figure 5) retinitis pigmentosa [87], X-linked retinoschisis [88], myotonic dystrophy, Alport syndrome [89], Coats’ disease [90], high myopia [91,92,93,94], proliferative diabetic retinopathy [95], familial exudative vitreoretinopathy [96] and ocular trauma [97,98] may experience a spontaneous dehiscense of the roof of a cystoid space causing a LMH. This dehiscense may be triggered by vitreomacular traction by either an epiretinal membrane or the posterior hyaloid [78,99,100]. Macular telangiectasia type 2 (MacTel2) may also be associated with an LMH. MacTel 2 is characterized by Müller cell dysfunction. Death of Müller cells can cause tissue loss that leads to a LMH [101]. Eyes with myopic tractional maculopathies may also progress to LMH.

Degenerative LMH is a chronic and progressive condition. Its pathogenesis is poorly understood [16,17]. Many investigators suggested that foveal remodeling in the absence of overt tractional forces are at play [17,27,44,46,47]. However, according to Bringmann et al. [64,72], the initial event in both ERM foveoschisis and LMH is a tractional event that disrupts the Müller cell cone in the foveola or the connections to the foveal walls. This tractional event creates a schisis between the OPL and HFL. Cavitations emerge and enlarge by a slow and chronic degeneration of Henle fibers [64]. Continuous chronic degeneration of HFL coupled to an unknown event such as choroidal ischemia may lead to degenerative changes of the retinal outer layers causing central photoreceptor death and retrograde degeneration of bipolar and horizontal cells [64,72,102].

## 7. Natural History

The natural history of LMH is poorly understood. LMH may close spontaneously with extension of the ERP size over time [103,104,105,106]. ERP most likely represents Müller cell proliferation as a reactive process to retinal injury [46,48,106]. Conversely some eyes with a LMH may develop into a FTMH. Most of the eyes that converted from a LMH to a FTMH had an ERM and/or ERP suggesting that tangential traction could play a role in this situation [44,46,49,77,107]. Alternatively, a poorly understood progressive degeneration of the foveolar architecture could be responsible for the progression to a FTMH. In these cases, the macular hole diameter was typically small, the edges were flat and there was limited retinal hydration. Although most FTMH closed following surgical repair, the resulting visual acuities were relatively poor [52].

Some observational case series suggest that LMH are relatively stable conditions [47,93,108,109,110]. Forty one eyes were followed for an average of 37 months with time domain OCT. LMH were diagnosed according to the following four criteria: irregular central foveal thinning, opening of the inner foveal layers, intraretinal splitting and intact foveal photoreceptors [8,25,108]. Over this time the diameter of the LMH increased by 14%. The BCVA remained stable in 78% and worsened in 22%. The foveal thickness decreased by an average of 10.3% during this period of time as well [108]. A small case series of 34 eyes were followed for an average of 18 months with SD-OCT. Most of these eyes remained stable. However, two eyes developed a FTMH [44]. The presence or absence of ERP does not appear to play a role in the progression of LMH [44,47]. Another retrospective study of 46 eyes that were followed for at least 5 years revealed that eyes with LMH, as defined by the International Vitreomacular Traction Study Group [2], MPH and MPH with cleaved edges [31] remain stable. A recent retrospective longitudinal case series, which used the recent classification proposed by Hubschman et al. [17], reported that visual acuity remained stable in eyes with ERM foveoschisis, LMH and MPH. These eyes had a mean follow up of 10, 11 and 19 months, respectively [109].

On the other hand, others have shown that LMH are not static entities and may undergo changes with time [15,108,111]. Theodossiadis and colleagues [108] reported that in their series of 41 eyes that were followed for a mean of 37 months with time domain OCT, 22% of eyes developed a decrease of visual acuity from baseline. The visual loss was associated with an enlarged LMH diameter, decreased foveal thickness and photoreceptor layer integrity [108]. A retrospective longitudinal study of 189 eyes with LMH showed progressive enlargement of the maximal diameter of the intraretinal splitting over 12–24 months of follow-up [15]. Another retrospective longitudinal study compared 28 eyes with LMH and 21 healthy control eyes. Eyes were assessed with multifocal ERG and SD-OCT at baseline and at 12 months of follow-up. At 12 months of follow-up almost a third of LMH eyes experienced morphological deterioration, evidenced by a mean decrease of 10% in central retinal thickness [111].

## 8. Treatment

Surgical intervention for LMH remains controversial and no clear guidelines exist for pars plana vitrectomy (PPV). Invariably many of the early series included both ERM foveoschisis and LMH eyes without differentiating between these two different conditions. Some authors suggest that there is no evidence that justifies PPV [8,10,11], while others feel that vitrectomy should be reserved for cases where the visual acuity continues to decrease or when the macula experiences progressive macular thinning [27,112]. Others have shown that PPV with ERM and ILM removal improves the visual and anatomic outcomes [10,12,13,113,114,115,116,117]. PPV with air or gas tamponade may lead to improved functional and anatomic outcomes [13,117], Presumably peeling the ERM and the ILM releases tangential traction to the edges of the LMH [117]. Garretson and colleagues [12] reported on their series of 27 eyes with a symptomatic LMH that underwent PPV with ERM and ILM peeling. After a mean follow-up of 9 months, 93% of eyes improved their baseline visual acuity with a mean gain of 3.2 Snellen lines. One eye developed a FTMH post-operatively and despite surgical repair the patient ended up with a loss of visual acuity when compared to baseline. Another patient lost a line of visual acuity despite cataract extraction and post-operative normalization of the macular morphology on OCT [12]. Taiwanese researchers reported that in eyes with LMH associated with an ERM, PPV with ERM and ILM peeling improved the visual outcomes [113]. LMH were defined by the criteria of Witkin et al. [9]. Eyes with an MPH were specifically excluded. Indications for PPV included a BCVA ≤ 20/40 and the presence of an ERM. Thirty eyes met the inclusion and exclusion criteria. They found that eyes subjected to gas tamponade had a better restoration of the macular configuration, but the post-operative visual acuity was related to an intact ellipsoid zone rather than the macular configuration. Eyes gained an average of 3.4 Snellen lines after PPV. One eye developed a post-operative FTMH which was successfully repaired [113]. Some believe that gas tamponade and prone positioning is an important adjunct [114,115]. In contrast others believe that fluid air exchange and positioning is not necessary [12,28,113,118]. Garretson et al. [12] reported 5 eyes that did not undergo fluid air exchange and equally gained visual acuity when compared to those that underwent fluid air exchange and gas tamponade.

Authors of more recent contemporary publications have adopted the newer LMH nomenclatura [16,17]. Eyes with a tractional LMH that underwent PPV, ERM peel and gas tamponade experience good anatomical and functional success rates [12,27,116,119]. A recent systematic review and meta-analysis reported the surgical outcomes of 463 eyes from 13 studies. Patients were subdivided into the following three groups: degenerative LMH, LMH with ERP and tractional LMH. All groups experienced a similar improvement in visual acuity following surgical repair with PPV. However, eyes in the degenerative LMH and LMH with ERP groups had a higher incidence of post-operative FTMH [120]. Obata and co-workers [121] reported that PPV improved visual acuity in both tractional LMH and degenerative LMH. Furthermore, there was no difference in the degree of improvement in visual acuity in both groups. In contrast other authors reported that the post-operative visual acuity improved in tractional LMH but not in degenerative LMH [49,61,62,122]. A recent Cochrane review could only assess a single randomized clinical trial for surgical intervention for LMH, underscoring the lack of evidence for surgical intervention for LMH [123,124].

ERM and ILM peeling may lead to a FTMH, particularly in eyes with degenerative LMH with ERP [11,12,16,27,44,46,115]. Surgical modifications to improve the outcomes of degenerative LMH and to avoid iatrogenic FTMH, include the double inverted flap technique [125], ERP embedding into the retinal cleavage technique [126], ERP embedding combined with fovea sparing ILM peel [127], and highly concentrated autologous platelet-rich plasma with ILM peeling [128,129]. A recent small, randomized prospective study compared the outcomes of eyes with degenerative LMH and ERP that underwent PPV with foveal sparing ILM peeling and observation [124]. They reported that at 6 months of follow-up, the eyes in the surgical arm experienced an improved foveal retinal sensitivity, improved BCVA and increased central retinal thickness when compared to the eyes in the observation arm.

Frisina and colleagues [125] compared the outcomes following ERM and ILM peel vs. an inverted double flap of ERM and ILM in eyes with ERP and LMH. Eyes that underwent the inverted double flap technique fared better than eyes that underwent ERM and ILM peeling. On average the BCVA improved whereas there was no improvement on average in the eyes treated with ERM and ILM peel. Furthermore 3/18 eyes developed an FTMH. Several groups have reported that complete ILM peeling across the macular area leads to a greater disruption of the ellipsoid zone and worse post-operative visual acuity in eyes with LMH and ERP compared to eyes without ERP [61,62,113]. Some surgeons recommend ILM peeling with sparing of the fovea and filling the macular defect with ERP [127].

Several pre-operative biomarkers, including pre-operative visual acuity, foveal thickness, ellipsoid zone integrity and the presence of ERP, have been explored as post-operative prognostic factors [62,130,131]. A poor pre-operative visual acuity (≤20/100), a thin fovea (≤100 µm), pre-operative ellipsoid zone disruption, absence of vitreopapillary adhesion or the presence of ERP were identified as poor prognostic signs for LMH undergoing surgical repair [62,130,132].

The effect of the presence of ERP on the surgical outcomes of LMH remains unclear. A meta-analysis that included eight studies and 350 patients compared the surgical outcomes of eyes with LMH with or without ERP. In all studies the pre-operative BCVA between the two groups was not statistically different. BCVA improved in both groups but the eyes without ERP had a better post-operative BCVA [133]. Several studies report that there was no surgical benefit for eyes with ERP [59,61,62,122]. Lai et al. reported that 75% of eyes had a similar improvement in BCVA regardless of the presence of ERP. ERP is associated with poorer functional and anatomic outcomes following surgical repair of FTMH [51]. Ko and colleagues [62] compared the surgical outcomes of eyes with LMH and ERP to eyes without ERP. At baseline both groups had a similar BCVA. After a mean follow-up of 21.5 months, they reported that the BCVA improved from 0.33 logMAR to 0.10 logMAR. In contrast, eyes with ERP the BCVA remained unchanged at 0.33 logMAR. They concluded that there was no surgical benefit for eyes with ERP [62]. In contrast, other investigators report that ERP did not affect surgical outcomes [27,49,53,55,131].

In summary, eyes with symptomatic ERM foveoschisis and eyes with LMH characterized by progressive thinning may benefit from surgical repair. 

## 9. Conclusions

Macular conditions characterized by an irregular foveal contour caused by a break in the inner fovea were recognized and classified by slit-lamp biomicroscopy. The advent of SD-OCT allowed a clear differentiation between FTMH and other simulating conditions. However, the diagnostic criteria for LMH have evolved over the past two decades. The latest consensus definition tries to distinguish conditions secondary to a tractional pathogenesis to those from a degenerative pathogenesis. Recent observations, however, suggest that we have come full circle. Both ERM foveoschisis and LMH appear to represent different phenotypes of a tractional disruption of the foveolar Müller cells. Eyes with documented progressive visual loss or morphological deterioration of the foveal architecture may be candidates for surgical repair.

## Data Availability

Not applicable.
